# Atraumatic Occult Odontoid Fracture in Patients with Osteoporosis-Associated Thoracic Kyphotic Deformity: Report of a Case and Review of the Literature

**DOI:** 10.1155/2015/301858

**Published:** 2015-08-26

**Authors:** Kanji Mori, Kazuya Nishizawa, Akira Nakamura, Shinji Imai

**Affiliations:** Department of Orthopaedic Surgery, Shiga University of Medical Science, Tsukinowa-cho, Seta, Otsu, Shiga 520-2192, Japan

## Abstract

Anderson type II odontoid fractures are reported to be the most common injury of the odontoid process in patients over the age of 65. However, atraumatic occult Anderson type III odontoid fractures have been rarely described and remain a diagnostic challenge. In the present report, we illustrate a 78-year-old female with osteoporosis-associated marked thoracic kyphotic deformity who developed atraumatic Anderson type III occult odontoid fracture and raise awareness of this condition. Anteroposterior and lateral standard radiographs of cervical spine failed to disclose odontoid fracture. Magnetic resonance imaging demonstrated intensity changes of the axis. Subsequent computed tomography clearly demonstrated Anderson type III odontoid fracture. Conservative treatment achieved complete bone union without neurological deteriorations. At 3-year follow-up, the patient was doing well without neurological and radiological deteriorations. Even if the patients have no traumatic event, we have to keep odontoid fractures in our mind as one of the differential diagnoses when we encounter elderly patients with neck pain, especially in patients with osteoporosis-associated marked thoracic kyphotic deformity.

## 1. Introduction

Odontoid fractures are caused by minor trauma like falls from standing or seated height in elderly individuals [1]. Type II odontoid fracture classified by Anderson and D'Alonzo [2] is the most frequent individual fracture of the cervical spine in elderly patients [1]. Anderson type III odontoid fracture is the second one, most resulting from traumatic events [2, 3]. Occult odontoid fractures without any traumatic event have been rarely described in the literature and remain a diagnostic challenge [3].

In the present report, we illustrate a unique case of elderly female patients with occult Anderson type III odontoid fracture without any traumatic event. We emphasize the importance of an awareness of this condition and discuss the likely mechanism of the injury in the present case.

## 2. Case Presentation

**Figure 1:**
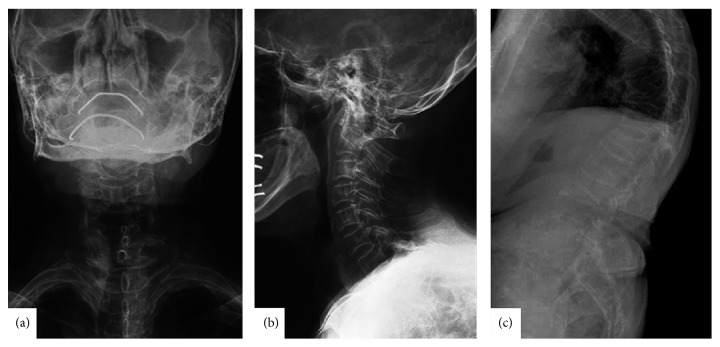
Anteroposterior (a) and lateral (b) standard radiographs of cervical spine revealed degenerative spondylosis but failed to reveal apparent fracture in cervical spine. Lateral standard radiographs of thoracolumbar spine revealed marked thoracic kyphotic deformity due to multiple compression fractures (c).

A 78-year-old female experienced sudden severe neck pain when she looked up the refrigerator (her cervical spine was extended) to open its door and visited our institution. She had a mild occasional neck pain before this event; however she reported no traumatic event just before the onset of this symptom or in the past. Physical examination revealed the absence of neurological compromise. In turn, anteroposterior and lateral standard radiographs of cervical spine revealed cervical degenerative spondylosis, but we could not detect apparent fracture (Figures 1(a) and 1(b)). Lateral standard radiographs of thoracolumbar spine revealed marked thoracic kyphotic deformity due to multiple compression fractures (Figure 1(c)). It was difficult to find normal-shaped vertebrae in thoracic spine and thoracic kyphosis angle measured by T5-12 was 75 degrees (Figure 1(c)). Her bone mineral density (*T*-score: −3.5) was significantly low.

**Figure 2:**
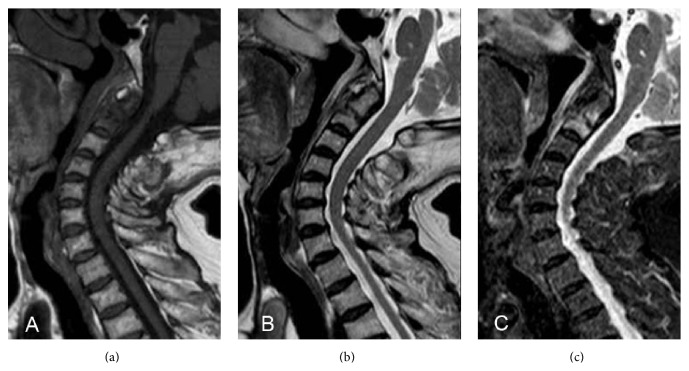
Magnetic resonance imaging demonstrated overt intensity changes of C2, which was low intensity on both (a) T1- and (b) T2-weighted images but high intensity on STIR (c) images. Spinal cord involvement was not evident.

**Figure 3:**
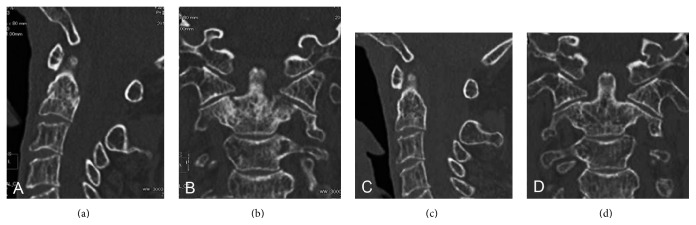
Sagittal (a, c) and coronal (b, d) reconstruction images of computed tomography (CT). (a, b) Anderson type III odontoid fracture was confirmed at the time of initial diagnosis. (c, d) Follow-up CT obtained after 6-month conservative treatment clearly revealed complete bone union.

She complained about persistent severe diffuse neck pain. Four days later, magnetic resonance (MR) imaging was performed to rule out latent fresh compression fracture and overt intensity changes of axis, which was low intensity on both T1- and T2-weighted images but high intensity on STIR image, were found (Figures 2(a)–2(c)). Subsequent computed tomography (CT) clearly demonstrated Anderson type III odontoid fracture (Figures 3(a) and 3(b)). Routine blood tests including serum Ca, P, and parathyroid hormone level were unremarkable.

After the careful explanation of the risk of nonunion/malunion and neurological deteriorations, the patient refused surgical treatment as well as Halo-vest fixation. Conservative treatment with Philadelphia type cervical caller was indicated. Two-month conservative treatment with cervical caller achieved good pain relief. Follow-up CT after 6 months from the initial treatment revealed significant bone union (Figures 3(c) and 3(d)). No apparent instability was found by dynamic lateral standard radiographs of cervical spine (data not shown). At the latest follow-up, 3 years after the onset, the patient was doing well without neurological and radiological deteriorations.

## 3. Discussion

Anderson and D'Alonzo classified odontoid fractures into 3 types based on the localization of the fracture line passed through [2]. Type I fractures are oblique fractures through the upper portion of the odontoid process; type II fractures cross the base of the odontoid process at the junction with the axis body; and type III fractures extend deep through the cancellous portion of the body of axis at the base of the dens. In elderly individuals, odontoid fractures are not uncommon [1, 2]. In patients over the age of 65, type II odontoid fractures are the most common injury of the odontoid process [1, 4–7].

Several studies revealed that, compared with patients younger than 65 years, elderly patients require lower energy, that is, fall from standing or seated height, to fracture their cervical spines [4–7]. Atraumatic occult odontoid fractures like our case are rarely described and remain diagnostic challenge. Only a few such cases were described previously [3, 8–10].

Odontoid fractures are mostly induced by nonphysiological flexion, extension, or rotation force of the upper cervical spine; however exact mechanism is not fully determined [3, 11]. Senescent degenerative changes tend to occur in the mid- and lower cervical spine, shifting a greater degree of mobility onto the C0 through C2, which is considered as one of the reasons why fractures of upper cervical spine often occur in elderly [4–6]. However, to the best of our knowledge, there is no report that discussed the influence of sagittal malalignment of the spine on the odontoid fracture.

The precise mechanism of odontoid fracture in the present case remains unknown; however significant osteoporosis might play a pivotal role on our case. In addition to significant osteoporosis, we advocate the possible influence of sagittal malalignment of the spine on odontoid fracture in this case. Namely, a compensatory hyperlordosis of cervical spine due to marked thoracic kyphotic deformity might yield routine hyperextension stress to the cervical spine. In turn, senescent degenerative changes make the mid- to lower cervical spine stiffer and shift the motion segment to the upper cervical spine [1]. These altered biomechanics might cause repeated hyperextension stress on the upper cervical spine including axis. It is likely that the combination of altered routine stress to the upper cervical spine and osteoporosis might ultimately result in Anderson type III odontoid fracture when the patient looked up the refrigerator in the present case.

Standard radiographs are routinely used as a screening tool for evaluating cervical spine injuries; however they could not always detect them [3, 12, 13]. Thus, several authors have advocated CT screening for the evaluation of cervical spine fracture in high-risk patients [1, 3, 13]. Consistent with previous reports, CT clearly revealed the odontoid fracture in the present case. We advocate that the elderly patients with osteoporosis-associated marked thoracic kyphotic deformity should be included in high-risk patients of odontoid fractures. If such patients visit hospital, that is, emergency department with neck pain, we recommend performing CT examination to rule out latent fracture even if the patient has no traumatic event.

The optimal management of odontoid fractures in the elderly population remains unsettled and management of this group of patients is complicated by multiple comorbidities [2, 14–16]. Pepin et al. have reported that nonunion rate reached 15% in type III odontoid fractures and advocated surgical treatment [15]. Recently, Patel et al. reported high rates of bony union and stable fibrous nonunion with a good functional outcome can be achieved in the elderly population sustaining type II or III odontoid fractures and advocated conservative management [16]. Taking previously reported favorable results of conservative treatment for this type of fracture [2, 14, 16] into account, we selected conservative treatment with Philadelphia type cervical caller in the present case.

In conclusion, even if the patients have no traumatic event, we have to keep odontoid fractures in our mind as one of the differential diagnoses when we encounter elderly patients with neck pain, especially in patients with osteoporosis-associated marked thoracic kyphotic deformity.
